# IRAP Inhibitors: M1-Aminopeptidase Family Inspiration

**DOI:** 10.3389/fphar.2020.585930

**Published:** 2020-09-25

**Authors:** Nicholas Barlow, Philip E. Thompson

**Affiliations:** Medicinal Chemistry, Monash Institute of Pharmaceutical Sciences, Monash University, Parkville, VIC, Australia

**Keywords:** insulin regulated aminopeptidase, aminopeptidase, enzyme inhibitor design, transition state analog, small molecule inhibitor, peptidomimetic

## Abstract

The insulin regulated aminopeptidase (IRAP) has been proposed as an important therapeutic target for indications including Alzheimer’s disease and immune disorders. To date, a number of IRAP inhibitor designs have been investigated but the total number of molecules investigated remains quite small. As a member the M1 aminopeptidase family, IRAP shares numerous structural features with the other M1 aminopeptidases. The study of those enzymes and the development of inhibitors provide key learnings and new approaches and are potential sources of inspiration for future IRAP inhibitors.

## Introduction

The insulin regulated aminopeptidase (IRAP) is a zinc-dependent M1 aminopeptidase and a type II transmembrane protein with a cytoplasmic N-terminal domain and an extracellular/intra-endosomal C-terminal domain containing the catalytic zinc domain (Keller et al., 1995; Keller et al., 2002). IRAP is found in almost all human tissues (Keller et al., 1995; Tsujimoto and Hattori, 2005) and is known to be expressed in a range of neuronal cells (Fernando et al., 2005), placental cells (Nomura et al., 2005), and leukocytes (Saveanu et al., 2009). IRAP appears to play three distinct physiological roles. Firstly, IRAP degrades a number of extracellular signaling peptides through the removal of the N-terminal amino acid. Proposed substrates include oxytocin, vasopressin, angiotensin III, Met-enkephalin, dynorphin A, neurokinin A, neuromedin B, somatostatin, and CCK-8 (Rogi et al., 1996; Herbst et al., 1997; Matsumoto et al., 2001; Lew et al., 2003; Tsujimoto and Hattori, 2005), although the physiological relevance of these remains controversial. Secondly, IRAP participates in MHC class I antigen presentation through amino terminal trimming of exogenous cross-presenting peptides (Saveanu et al., 2009; Saveanu and van Endert, 2012). Thirdly, IRAP is co-located with Glut4 in insulin-responsive membrane vesicles and is thought to play a role in the insulin-induced translocation of these vesicles to the plasma membrane thus regulating cellular glucose uptake (Waters et al., 1997; Bryant et al., 2002; Pan et al., 2019). Interestingly, genetic deletion of IRAP in mice has not been associated with any major health defects, but rather provides protection against damage due to cerebral ischemia (Pham et al., 2012), thrombosis (Numaguchi et al., 2009; Gaspari et al., 2018) in models of those respective injuries, as well as diet-induced obesity (Niwa et al., 2015).

**Figure 1:**
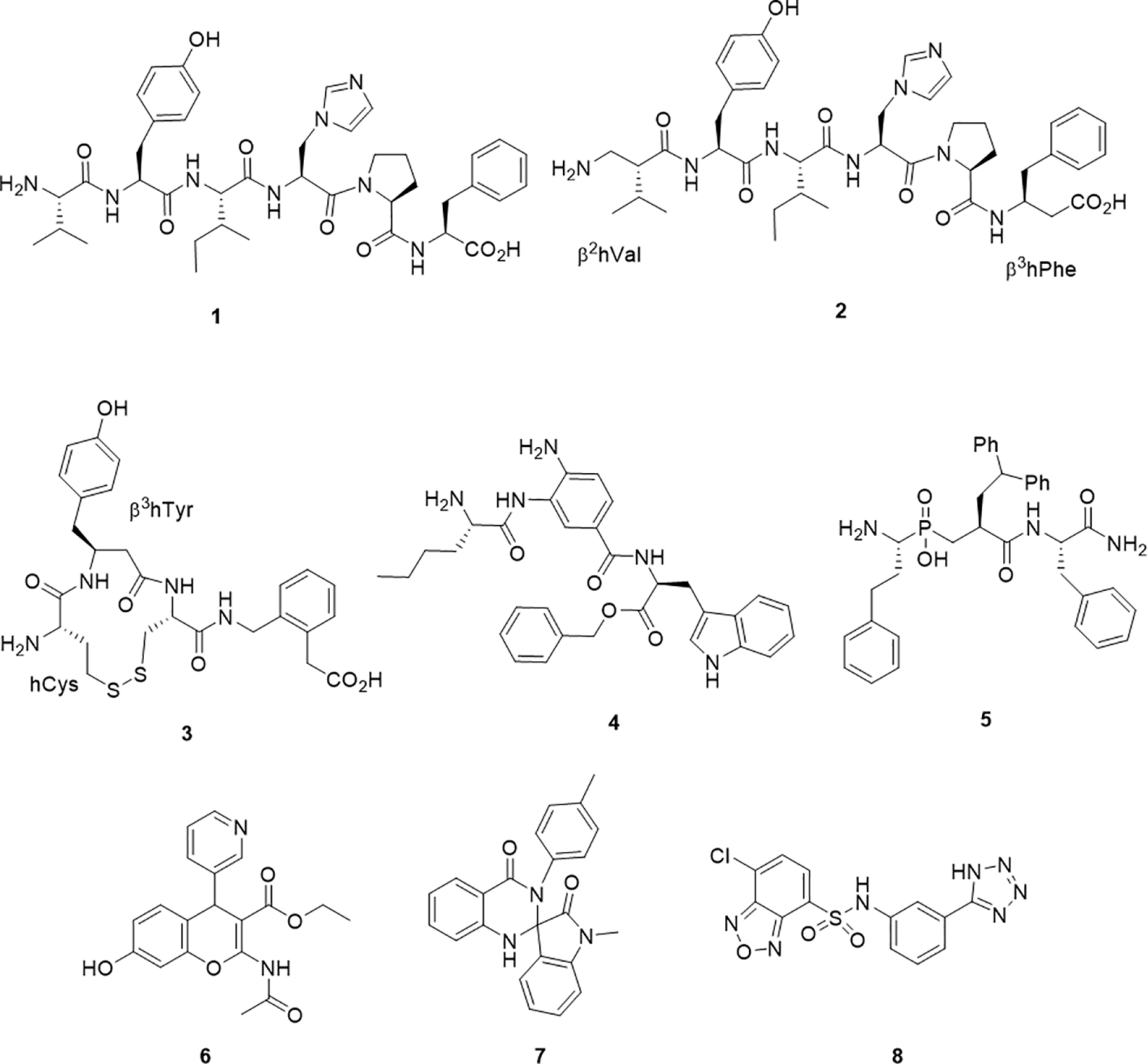
Chemical structures of IRAP inhibitors.

Commensurate with this pleiotropic character, IRAP is a potential therapeutic target for a range of therapeutic applications. In particular, IRAP is a potential therapeutic target for the treatment of Alzheimer’s disease and other cognitive impairments. Rodents treated with the IRAP inhibitors such as Angiotensin IV (AngIV, 1) (**Figure 1**) *via* intracranial injection or subcutaneous administration, show improved performance in learning and memory (Wright et al., 1999; Albiston et al., 2001; Pederson et al., 2001; Lee et al., 2004; Gard, 2008; Golding et al., 2010). The cellular mechanism for this increased learning is unclear and may be attributable to modulation of glucose uptake *via* GLUT4 containing vesicles or reduced degradation of oxytocin and vasopressin, which have both been shown to improve learning and memory (Vanderheyden, 2009; Fidalgo et al., 2019). A second emerging therapeutic application is the potential of IRAP inhibitors to protect against stroke, thrombosis, and obesity-related disorders in comparable fashion to the knockout phenotypes. A third therapeutic potential for IRAP inhibitors is drawn from their role in preparing peptides for cross-presentation. Disruption of this function by inhibitors has been demonstrated *in vitro* and underscores their potential application in cancer immunotherapy or control of autoimmunity (Stratikos, 2014; Papakyriakou et al., 2015; Kokkala et al., 2016). Together, this pool of research suggests that there may be a number of indications for IRAP inhibitors.

## A Quick Snapshot of IRAP Inhibitor Development

The search for inhibitors of IRAP dates back to its discovery as oxytocinase in 1959 (Hooper, 1959). In this first study of serum aminopeptidase activity from human placentae, Cu^2+^, di-isopropylphosphofluoridate (DFP), tetraethylpyrophosphate (TEPP), and ethylenediamine tetra acetic acid (EDTA) were shown to block the enzyme activity, signaling the metalloprotease nature of the enzyme. The first attempts to block this with competing ligands is described soon after using modified oxytocin peptides (Berankova et al., 1960; Berankova and Sorm, 1961). However, further progress appears to have been hampered by the challenges of the complex protease mixtures, including other M1 family members, in the tissue sources being studied.

A major step forward was the identification of the hexapeptide, AngIV, 1 (1) as an IRAP inhibitor. The memory enhancing effects of Ang IV administration and as well as its inhibitory effect on oxytocin metabolism were both reported and investigated separately prior to the appreciation that these effects were modulated through the action of a single target – IRAP (Braszko et al., 1988; Albiston et al., 2001). Ang IV is a component of the renin-angiotensin system as a degradation product from the proteolytic truncation of the vasoconstricting peptide, angiotensin II. With good affinity but poor plasma stability, successful structural modifications of 1 have led to the β-amino acid containing peptide mimetic 2 (*K*_i_ = 27 nM), which contains both N and C terminal β-amino acids and is less susceptible to degradation by IRAP (Lukaszuk et al., 2008) and the analogue IVDE77 (Nikolaou et al., 2013). In an alternate but comparable peptidomimetic approach, cyclic compounds, HA-08, 3 (*K*_i_ = 3.3 nM) and analogs were developed (Andersson et al., 2010). The design of these compounds had in mind IRAP’s unique ability to process cyclic peptides like oxytocin and vasopressin as part of the design. HA-08 was recently co-crystallised with IRAP (Mpakali et al., 2020), and a considerable body of SAR data pertaining to the cyclic structure has been accumulated (Andersson et al., 2011; Barlow et al., 2020).

Other IRAP inhibitors have been developed from investigations into the S1 subsite using fluorogenic substrates. Non-natural amino acids such as homoPhe and Nle were identified as S1 residues that conferred some substrate selectivity over other M1 aminopeptidases, ERAP1 and ERAP2 (Zervoudi et al., 2011). The selectivity of these probes inspired the development of the aminobenzamide inhibitor 4, which has good potency (IC_50_ = 110 nM) and selectivity against ERAP1 and ERAP2 (Papakyriakou et al., 2015). Transition state mimetics which build on the growing understanding of P1 and P1` SAR have also been effective. In particular, a number of phosphinic acids including 5 have exhibited good potency (Kd = 18 nM) (Kokkala et al., 2016). Compound 5 was also the first inhibitor to be co-crystallised in complex with IRAP (Mpakali et al., 2017a).

The first the small molecule inhibitor series to be described were benzopyrans discovered by a virtual screening approach, including HFI-419, 6 (Albiston et al., 2008; Mountford et al., 2014). This molecule also displays good potency (*K*_i_ = 0.48 µM). More recently, high throughput screening approaches have led to the discovery of 7 (IC_50_ = 6.1μM) and 8 (IC_50_ = 0.4μM) (Engen et al., 2016; Vanga et al., 2018).

While this represents a diverse series of compounds that have played their roles in defining the pharmacology of IRAP inhibitors, none have emerged as *bone fide* drug candidates as yet. In part, the challenges of delivery to the CNS for indications such as Alzheimer’s disease have hampered the progression of peptide-like molecules and have also proved challenging for small molecules. Similarly, for immunological antigen processing, intracellular delivery will be a requirement. Advancing these, or future series of IRAP inhibitors, will therefore require close attention to the specific requirements related to each indication.

## Inspirational Inhibitors Within the M1 Aminopeptidase Family

The human M1 aminopeptidase family, which includes IRAP, contains 12 members (**Table 1**) (Rawlings et al., 2014). All members utilize a single catalytic zinc atom in a conserved HExxHx18E motif and contain a substrate binding domain comprised of a conserved GxMEN motif. Structural similarities between M1 aminopeptidases result in a number of common substrates that are degraded by more than one family member. Indeed, the N-terminal specificity of M1 aminopeptidases in known to be broad and overlapping, and inhibitors are usually required to engage several subsites in order to achieve selectivity (Rawlings and Barrett, 2013).

**Table 1:** M1 aminopeptidases.

M1 Aminopeptidases	Abbreviation	S1 substrates	Publications*	X-ray structures^#^
Insulin Regulated Aminopeptidase	IRAP	C, L, K, R, M	170	5MJ6 (Mpakali et al., 2017a), 4PJ6 (Hermans et al., 2015)
Aminopeptidase N	APN	A, F, Y, L, P	1700	4FYS (Wong et al., 2012), 6BV3 (Joshi et al., 2017)
Aminopeptidase A	APA	E, D	515	4KXB (Yang et al., 2013)
Leukotriene A_4_ Hydrolase	LA4H	A, R, L	486	6ENB (Numao et al., 2017), 605H (Lee K. H. et al., 2019)
Thyrotropin-releasing hormone-degrading ectoenzyme	TRHDE	pGlu	9	
Puromycin-sensitive aminopeptidase	PSA	A, L, K	109	
Arginyl aminopeptidase N	APB	R, K	111	
Endoplasmic reticulum aminopeptidase 1	ERAP1	L, M, C, F	128	6T6R (Liddle et al., 2020), 6RYF (Giastas et al., 2019)
Endoplasmic reticulum aminopeptidase 2	ERAP2	R, K, M	31	5K1V (Mpakali et al., 2017b)
Arginyl aminopeptidase like 1	RNPL1	A, L, S, L, M	20	
Aminopeptidase Q	APQ	L, R, K, M	13	
Aminopeptidase O	APO	R, N	4	

*Publications in PubMed that contain the M1 aminopeptidases name in the title, abstract or text.

^#^Recent examples of crystal structures deposited in the Protein Data Bank (PDB).

The collected crystallographic data (**Table 1**) supports the common structural features of the M1 family as well as various archetypes that distinguish them. A conserved tyrosine residue typically binds the distal C-terminal, and interdomain movement may allow binding of diverse substrates (Cadel et al., 2015; Drinkwater et al., 2017). In particular, both closed and open conformations of the catalytic domains are observed. A dynamic model has been proposed whereby the domains II and IV close around extended substrates to support binding and hydrolysis (Mpakali et al., 2020). This mode of binding may be mimicked by inhibitors, but the dynamic nature provides a challenge to structure-based inhibitor design.

Other members of the M1 aminopeptidase family have also been identified as targets for therapy, most notably APN, APA, LTA4H, and ERAP1. Given the similarities of the family members, it seems that the study of other inhibitor designs within the M1 aminopeptidase family may provide interesting insights into inhibition mechanisms that are pertinent for inhibitor design and could be exploited to provide new IRAP inhibitors.

## Isoform Hopping—Endoplasmic Reticulum Aminopeptidase 1

**Figure 2:**
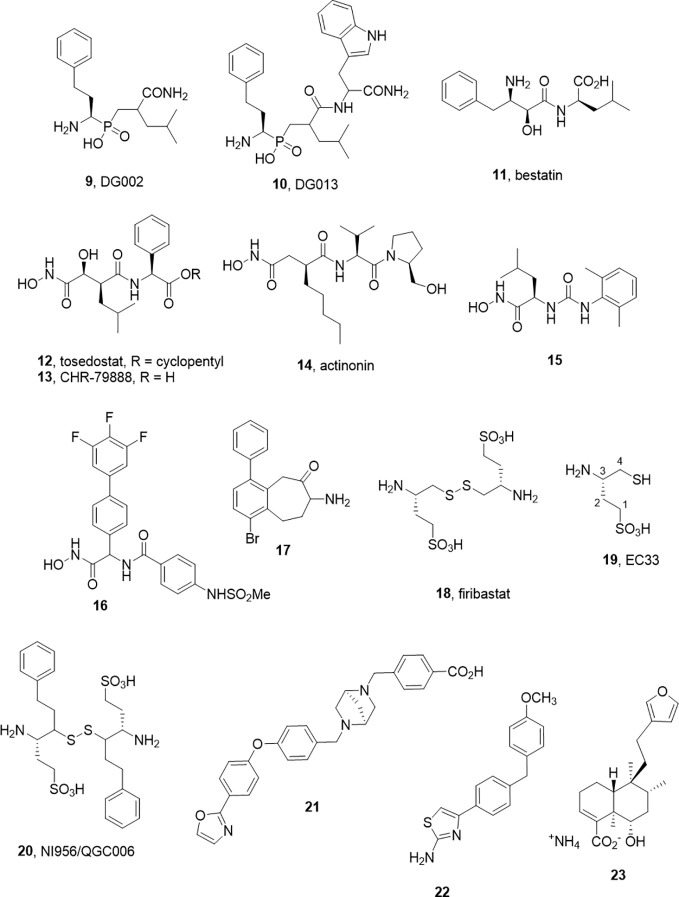
M1 aminopeptidase inhibitors from other families.

As alluded to above, inhibitors of one aminopeptidase class can be expected to inhibit other classes. In a pessimistic sense, this characterizes the challenge of achieving selectivity, while in a positive sense, redesigns can be used to tune selectivity between family members without major changes to the core molecular scaffold. This selectivity transitioning often manifests within derivative libraries. For example, Zervoudi et al. identified the phosphinic acid inhibitors DG002 (9) and DG013 (10) to target antigenic processing enzymes, ERAP1, ERAP2, and IRAP (**Figure 2**) (Zervoudi et al., 2013). These compounds and, in particular, the *R,S,S*-diastereomer, DG013A, showed good potency across the three isoforms. The same team showed replacement of the central leucine yielded the IRAP selective compound 5 (DG026A) (Mpakali et al., 2017a).

Note that phosphinic acids have an even broader general history. As far back as 1989, such compounds were described as transition state analogs for metalloproteases (Grobelny et al., 1989; Yiotakis et al., 1994) and are represented in the M-17 Leucine amino peptidase inhibitors described in 2003 by Grembecka et al. (2003). The broader activity and/or selectivity of these ligands needs to be considered (Talma et al., 2019). It is an interesting feature that these peptidomimetic transition state analogs possess intracellular activity also (Koumantou et al., 2019).

## Success Is Hard to Achieve—Aminopeptidase N

As the archetype of the M1-aminopeptidase family, APN has been much studied and numerous attempts to develop inhibitors have been described. These have been extensively reviewed recently by Amin et al. (2018). As a protein, APN shares some features also characteristic of IRAP. They include the facts that APN is recognized to be involved in multiple functions (enzyme for peptide cleavage, and signalling molecule in signal transduction), it exhibits broad substrate specificity (although distinct from IRAP) and that it has been shown by crystallography to exist in an open and closed form (Joshi et al., 2017). It has been by far the most studied with respect to inhibitor development, although the results of those efforts are yet to yield an unambiguous drug candidate.

While a large body of work has accumulated in the development of APN inhibitors, the best known examples that have advanced to clinical studies, bestatin (11) and tosedostat (12), are potent but non-selective. Bestatin, bearing the pharmacophoric β-amino α-hydroxy amide motif was discovered as a natural product inhibitor. Tosedostat, is a synthetic product but shares the hydroxamic acid motif of the natural product actinonin (14) (**Figure 2**). While both bestatin and tosedostat’s acid metabolite, CHR-79888 (13), show good APN inhibition (IC_50_ ~ 200nM), the activity *in vivo* is not thought to derive simply from APN inhibition. Indeed, CHR-79888 is a potent LTA4 hydrolase inhibitor. A useful lesson to note regarding metabolism is that the circulating bioactive may have a much-altered selectivity profile compared to the administered drug.

Otherwise, these two compounds signal the generalised challenges of developing selective small aminopeptidase inhibitors. For active site binding aminopeptidases, the likely pharmacophore is built around S1 and S1’ binding and non-scissile interactions with the catalytic zinc atom. The compounds that achieve this typically will not be impeded from comparable interactions in other zinc metallopeptidase sites, rendering them non-selective. In developing substrate mimetic inhibitors the on-going challenge has been to tune down the generic zinc binding moiety (which can drive affinity) to exploit the subsite differences of across the metalloprotease families (Tsoukalidou et al., 2019).

The challenges associated with peptide-based analogs can imply replacement of backbone peptide bonds and the use of non-proteinogenic side chain motifs to enhance selectivity. For the former, the replacement of peptide bonds with ureido equivalents in the hydroxamic acid series led to some potent APN inhibitors such as 15. In the latter case, another series of hydroxamate based inhibitors exemplified by 16 (Ki 4.5 nM) were described by Lee J. et al. (2019). These molecules have no obvious peptide character and may offer improved opportunity for achieving selectivity.

Another small molecule series of interest are aminobenzosuberones, which have been identified as a scaffold that selectively inhibits mono-metallic aminopeptidases with 17 showing sub-nanomolar affinity against human APN (K_i_ = 350 pM) (Peng et al., 2017; Salomon et al., 2018). The origin of this series dates back to corresponding tetralones (Schalk et al., 1994) and the class appears to provide the advantages of small molecule inhibitors (low MW, potential oral availability, potentially BBB penetrating). Co-crystallization of a phenyl substituted benzosuberone with *Ec*PepN showed the binding poExxHx_18_E motifs with the ketone function present in an sp^3^ hydrated form, acting as transition state mimetic not dissimilar from the β-amino α-hydroxy motif of bestatin (Peng et al., 2017). The implications for IRAP inhibitor design in this class are evident with one example showing strong inhibition of IRAP also (K_i_ = 34 nM). One cautionary note is that these compounds, like many small molecules, are predicted to have challenging metabolism and toxicity profiles although this should be tested experimentally (Salomon et al., 2018).

## Designing for Selectivity and In Vivo Activity—Aminopeptidase A

In contrast, the development of the aminopeptidase A inhibitor, Firibastat (18) presents an optimistic picture of what is possible in the aminopeptidase class (Ferdinand et al., 2019). Firibastat has entered phase III clinical studies for therapy of treatment-resistant hypertension; yet at first view, it would seem an unlikely therapeutic. Firstly, the active species of Firibastat, (S)-3-amino-4-mercaptobutane-1-sulfonic acid (19, EC33) is a thiol. Thiols are known to be effective chelators of zinc and have been employed in a range of zinc enzyme inhibitors, most notably captopril. However, the capacity for numerous off-target chelating or covalent disulfide forming interactions would typically argue against pursuit of such compounds.

The discovery of Firibastat has a long history. Early work by Fournié-Zaluski et al. employed β-amino thiols as substrate mimetics targeting the S` subsite of APN (Fournie-Zaluski et al., 1992). An interesting comparison of zinc chelating groups within this report suggested that β-amino thiols are more effective inhibitors of APN than corresponding hydroxamic acid, phosphate, and carboxylate inhibitors. A compound from the series was found to have significant CNS activity when administered by iv injection as its disulfide prodrug form.

Turning their focus to APA, which cleaves N-terminal Glu or Asp residues and in particular cleaves the N-terminal Asp from angiotensin II, blocking angiotensin III formation. They found the acidic sulfonic acid derivative, EC33 was a potent and selective inhibitor (APA Ki = 0.37 µM, APN Ki = 25 µM) (Chauvel et al., 1994a; Chauvel et al., 1994b). Intracerebroventricular injection of EC33 was found to prevent APA production of the hypertensive agent angiotensin III, lowering central arterial blood pressure (Zini et al., 1996). By developing the corresponding disulfide prodrug 18, it has been possible to move to oral administration. The oral bioavailability is modest (10–15%), and the drug half-life is short (40 min) but with a somewhat heroic dose of 500 mg, twice daily Firibastat effectively reduced blood pressure in the cohort (Ferdinand et al., 2019). While these clinical studies continue, a second generation oral inhibitor NI956/QGC006 (20) with improved pharmaceutical properties is progressing (Keck et al., 2019).

## Non-Canonical Binding Site ligands—Leukotriene A4 Hydrolase

Leukotriene A_4_ hydrolase has been another M1 aminopeptidase that has attracted attention as a potential therapeutic target, particularly in inflammatory disease. As suggested by its name, the best studied feature of the enzyme is not its ability to cleave N-terminal amino acids form substrate peptides, but rather to hydrolyse the epoxide ring of Leukotriene A4 to Leukotriene B4 (Haeggstrom et al., 2007). LTB_4_ is an inflammatory lipid and thus blocking its formation would be considered a therapeutic option. However, the enzyme has also been shown to process a variety of peptides, most notably Pro-Gly-Pro a collagen degradation product which is a pro-inflammatory chemotactic peptide (Snelgrove et al., 2010). This implies that the action of LTA_4_H has opposing actions, which may be context dependent, and the opposing activities of LTA4H reside within distinct yet overlapping active sites, with specific amino acid residues required for each. Several inhibitors including Bestatin (11) and Tosedostat (12) have advanced to the clinics as well as Acebilustat (21) which is in phase 2 studies for treatment of cystic fibrosis (Bhatt et al., 2017). Clinical trial results to date have been disappointing which has led to the postulate that the inhibiting Pro-Gly-Pro degradation was countering the desired therapeutic effects. In response, the concept of biased inhibitors that spare the aminopeptidase activity has emerged including allosteric ligands (22) that activate Pro-Gly-Pro hydrolysis only (Lee K. H. et al., 2019) or those that block LTA_4_ hydrolysis and activate Pro-Gly-Pro hydrolysis.

By analogy, Liddle et al. recently described an allosteric ligand for ERAP1 (23) that activates hydrolysis of small substrates (such as Leu-AMC) while inhibiting cleavage of longer substrates by competing with the extended peptide binding site such as the antigen precursor, YTAFTIPSI. It is postulated to achieve this by stabilizing the dynamics of active site residues and/or facilitating conformational change to a partially closed, more active conformation (Liddle et al., 2020).

The actual benefit of these concepts remains controversial, especially in the case of the Pro-Gly-Pro-sparing LTA4H inhibitors (Numao et al., 2017), but the dual activity raises interesting questions for IRAP research. Firstly, have the full gamut of substrates for IRAP been examined? The physiologically relevant peptide substrates are still not confirmed for IRAP and it may also be that there are small peptides or other non-peptide substrates (e.g., lipids) that can be processed by this hydrolytic enzyme. Secondly, what signals might be associated with IRAP cleavage of peptide substrates? It could be the down-regulation of a bioactive substrate (such as vasopressin) or maybe the effects derive from the generation of a bioactive product.

## Conclusions

IRAP is emerging as a therapeutic target against a host of disease states. As well as long identified connection to indications such as cognition disorders due to the effects of AngIV, extended studies of gene deletion or modification are showing potential applications in stroke, thrombosis, and obesity-related disorders. The localization of IRAP in various tissues and cell types will also present other hypotheses proposing IRAP inhibitors as drug targets such as in immunotherapy and/or combatting autoimmunity.

As a member of the M1 aminopeptidase family, IRAP shares many structural and functional similarities with other family members that have been the subject of parallel drug discovery efforts. Much can be learned from these analogous drug discovery efforts that might be applied to IRAP inhibitor development and guide future drug discovery efforts. We have highlighted some of these efforts with in the M1 aminopeptidase family and outlined some of the success stories, insights, and interesting observations from these campaigns. We have also highlighted particular chemical scaffolds, which we feel may be adapted to serve IRAP focused drug discovery efforts. Hopefully, this information can foster progress in the development of candidate drugs that realize the therapeutic potential of IRAP inhibition.

## Author Contributions

NB and PT contributed equally to the research and authorship of the manuscript.

## Conflict of Interest

The authors declare that the research was conducted in the absence of any commercial or financial relationships that could be construed as a potential conflict of interest.
